# Pretransplant immune dysregulation and survival after HSCT in pediatric inborn errors of immunity

**DOI:** 10.70962/jhi.20260093

**Published:** 2026-10-19

**Authors:** Andrés Soffia, Arturo Borzutzky, Cristián Sotomayor

**Affiliations:** 1 Division of Pediatrics, Department of Infectious Diseases and Immunology, School of Medicine, Pontificia Universidad Católica de Chile, Santiago, Chile; 2 Division of Pediatrics, Section of Hematology, Oncology and Stem Cell Transplantation, School of Medicine, Pontificia Universidad Católica de Chile, Santiago, Chile; 3 Red de Salud UC CHRISTUS, Santiago, Chile

## Abstract

Allogeneic hematopoietic stem cell transplantation (HSCT) is curative for many inborn errors of immunity (IEIs), but the prognostic role of pretransplant immune dysregulation in pediatric patients remains unclear. We retrospectively studied 29 pediatric patients with genetically confirmed IEI undergoing HSCT between 2021 and 2025. Immune dysregulation was quantified using IDDA v2.1; HCT-CI and PARI were also calculated. 2-year overall survival (OS) was 79%, and event-free survival was 59%. Higher IDDA v2.1 scores were associated with inferior OS in univariate analysis (HR: 2.23 per five-point increase, P = 0.043) and remained associated in an exploratory multivariable model (HR: 2.76, P = 0.041). Harrell’s C-statistic for OS was 0.722 for IDDA v2.1, 0.661 for HCT-CI, and 0.772 for PARI. Exploratory quadrant analyses suggested that IDDA v2.1 provides complementary information, identifying patients classified as lower risk by comorbidity indices who nonetheless had adverse outcomes. These findings support IDDA v2.1 as an adjunct pretransplant risk stratification tool in pediatric IEI.

## Teaser

Soffia et al. show that pretransplant immune dysregulation, quantified using IDDA v2.1, predicts overall survival after HSCT in children with inborn errors of immunity. IDDA v2.1 provides complementary risk information beyond HCT-CI and PARI, identifying high-risk patients not captured by conventional comorbidity indices.

## Introduction

Inborn errors of immunity (IEIs) comprise a rapidly expanding and heterogeneous group of disorders, with the most recent International Union of Immunological Societies (IUIS) Expert Committee classification recognizing over 550 distinct entities involving 508 different genes and 17 phenocopies (1, 2). Beyond susceptibility to infections, immune dysregulation is now recognized as a central feature of IEI, affecting up to 26% of patients and encompassing autoimmunity, autoinflammation, lymphoproliferation, malignancy, and severe atopy (3, 4). It is estimated that more than 30% of genes currently associated with IEI are linked to primary immune regulatory disorders (PIRDs) (5). This paradigm shift is supported by large registry data showing that immune dysregulation may represent the initial manifestation in a significant proportion of patients, with up to two thirds presenting before the age of 6 years (4). Autoimmune and inflammatory complications not only contribute significantly to morbidity, but have also been associated with worse survival outcomes in IEI patients (3, 4, 6).

IEIs represent a substantial proportion of indications for hematopoietic stem cell transplantation (HSCT) in children (≈39% of nonmalignant transplants) and continue to show a growing trend, with increasing transplant activity reported in published European Society for Blood and Marrow Transplantation (EBMT) data (7). Allogeneic HSCT offers a feasible curative option for adaptive immune defects and for selected innate immune deficiencies (8, 9). In PIRDs, HSCT is frequently considered in the context of severe immune dysregulation refractory to conventional therapies, often involving multi-organ autoimmunity and significant treatment-related morbidity (6, 8, 9). Early intervention is usually critical to avoid irreversible organ damage from the disease or its treatment (8, 9, 10, 11). Importantly, disease severity at the time of HSCT is a key predictor of outcome. In particular, the presence of uncontrolled immune dysregulation and inflammatory activity has been associated with inferior posttransplant survival, emphasizing the need for adequate pretransplant disease control (6, 9, 11, 12). Similar findings have been reported in non-IEI settings, such as myelodysplastic syndromes, where pretransplant systemic inflammatory and autoimmune conditions have been identified as independent risk factors for mortality after HSCT (13).

Given the central role of immune dysregulation in IEI, tools to quantify disease activity have become increasingly relevant. The Immune Deficiency and Dysregulation Activity (IDDA) score was originally developed to assess disease burden in lipopolysaccharide-responsive and beige-like anchor protein (LRBA) deficiency, where lower pre-HSCT IDDA scores were associated with improved survival (14). More recently, the updated IDDA v2.1 score, comprising 21 parameters across a graded scale, has been applied across a broader spectrum of IEIs to capture and quantify systemic immune dysregulation (15). Pretransplant risk stratification in HSCT has traditionally relied on the Hematopoietic Cell Transplantation–Specific Comorbidity Index (HCT-CI), developed by Sorror et al. to quantify comorbidities as independent predictors of nonrelapse mortality and overall survival (OS) in adults (16). Given the recognized limitations of HCT-CI in the pediatric setting—where most adult-defined comorbidities are absent and the score provides limited risk discrimination—the Pediatric Adapted Risk Index (PARI) was recently proposed by Elfeky et al. as a more appropriate risk stratification tool for children undergoing HSCT (17). Fox et al. recently demonstrated in a cohort of adolescents and adults that both pretransplant IDDA v2.1 and HCT-CI scores significantly impacted OS and event-free survival (EFS) after HSCT. Importantly, they also identified a subgroup of patients classified as low risk by HCT-CI alone who exhibited substantial immune dysregulation by IDDA v2.1, highlighting the potential value of complementary pretransplant risk assessment (18). However, the predictive role of the pre-HSCT IDDA v2.1 score in posttransplant outcomes in children with IEI has not yet been explored. We therefore aimed to evaluate its association with post-HSCT outcomes and to explore its potential utility for risk stratification in the pediatric setting.

## Results

### Patient and transplant characteristics

A total of 29 pediatric patients with genetically confirmed IEI underwent allogeneic HSCT during the study period. All patients had complete clinical data available for analysis, and no patients were excluded. The median age at transplantation was 5.8 years (range 0.3–17.7), and 19 patients (65.5%) were male. The median follow-up after HSCT was 37.5 mo (95% confidence interval [CI]: 24.7–49.7 mo). The underlying IEI diagnoses included severe combined immunodeficiency (SCID) and combined immunodeficiency (48.3%), syndromic combined immunodeficiencies (10.4%), predominant antibody deficiencies (6.9%), immune dysregulation disorders (3.4%), phagocyte defects (10.4%), defects in innate immunity (13.8%), and bone marrow failure syndromes (6.9%). All patients had a genetically confirmed diagnosis. Detailed patient characteristics are summarized in Table 1.

**Table 1.** Patient and disease demographics

Characteristics	Cohort *n* = 29 (%)
**Age at transplant, median (years)**	**5.8 (range 0.3–17.7)**
**Sex, *n* (%)**	​
Male	19 (65.5)
Female	10 (34.5)
**Median follow-up after transplant (months)**	**37.5 (95% CI: 24.7–49.7)**
**Genetically confirmed diagnosis, ** ***n*** ** (%)**	**29 (100)**
**IDDA v2.1 score pre-HSCT, median (range)**	**8.5 (0–23.7)**

IEI subgroup and specific disease, *n* (%)	​	IDDA v2.1 score pre-HSCT per subgroup, median (range)
**SCID and CID**	**14 (48.3)**	**10.8 (3–23.7)**
CD40LG	6	5 (3–12.4)
RAG2 deficiency	2	10.8 (9–12.5)
IL-7Rα deficiency	1	14.5
CD3δ deficiency	1	23.7
DCLRE1C (Artemis) deficiency	1	13
ADA deficiency	1	8
DOCK2 deficiency	1	9.3
LAT deficiency	1	14
**CID with associated or syndromic features**	**3 (10.3)**	**8.6 (0–14.5)**
WAS	3	8.6 (0–14.5)
**Predominant antibody deficiencies**	**2 (6.9)**	**10.3 (8.5–12)**
Activated p110δ syndrome (APDS1)	1	12
NFKB2 deficiency	1	8.5
**Diseases of immune dysregulation**	**1 (3.4)**	**18.7**
IPEX syndrome	1	18.7
**Phagocyte defects**	**3 (10.3)**	**0 (0–10.5)**
Elastase deficiency (SCN1)	1	0
X-linked CGD	1	10.5
Leukocyte adhesion deficiency type 1 (LAD1)	1	0
**Defects in innate and intrinsic immunity**	**4 (13.8)**	**1.5 (0–3.5)**
GATA2 deficiency	3	0 (0–3)
STAT1-GOF	1	3.5
**Bone marrow failure**	**2 (6.9)**	**2 (1–3)**
DKCA2	1	1
DKCA3	1	3

Bridging therapy prior to HSCT, *n* (%)	7 (24.1)
Cyclosporine A	4 (13.8)
Rapamycin	2 (6.9)
Ruxolitinib	1 (3.4)

IEIs, inborn errors of immunity; SCID, severe combined immunodeficiency; CID, combined immunodeficiency; DCLRE1C, DNA cross-link repair 1C (Artemis); RAG2, recombination activating gene 2; IL-7Rα, interleukin-7 receptor alpha; CD3δ, CD3 delta chain; CD40LG, CD40 ligand; ADA, adenosine deaminase; DOCK2, dedicator of cytokinesis 2; LAT, linker for activation of T cells; APDS1, activated phosphoinositide 3-kinase delta syndrome type 1; NFKB2, nuclear factor kappa B subunit 2; IPEX syndrome, immune dysregulation, polyendocrinopathy, enteropathy, X-linked syndrome; SCN1, severe congenital neutropenia type 1; CGD, chronic granulomatous disease; LAD1, leukocyte adhesion deficiency type 1; STAT1-GOF, signal transducer and activator of transcription 1 gain-of-function; GATA2, GATA binding protein 2 deficiency; DKCA2, dyskeratosis congenita, autosomal recessive type 2; DKCA3, dyskeratosis congenita, autosomal dominant type 3.

Regarding donor source, 41.4% of patients received grafts from mismatched unrelated donors (MMUD), 37.9% from matched unrelated donors (MUD), 13.8% from haploidentical donors, and 6.9% from matched related donors (MRD). Stem cell sources included bone marrow (48.3%), peripheral blood stem cells (44.8%), and cord blood (6.9%). Conditioning regimens were predominantly treosulfan-based, most commonly treosulfan–fludarabine with or without thiotepa, in accordance with EBMT/European Society for Immunodeficiencies guidelines for HSCT in IEIs (9). According to standard conditioning intensity definitions, 20 patients (69.0%) received myeloablative conditioning (MAC), while 9 (31.0%) received non-MAC or reduced-intensity conditioning (RIC) regimens. For graft-versus-host disease (GVHD) prophylaxis, in vivo T cell depletion with anti-thymocyte globulin or alemtuzumab was used in all but one transplant, which involved an MRD. Detailed transplant characteristics, including conditioning regimens and GVHD prophylaxis, are summarized in Table 2.

**Table 2.** Transplant characteristics of the cohort

Characteristics	Cohort *n* = 29 (%)
**HCT-CI score, median (range)**	**1 (0–7)**
**HCT-CI category, *n* (%)**	​
Low (score 0)	12 (41.4)
Intermediate (score 1–2)	14 (48.3)
High (score ≥3)	3 (10.3)
**PARI score, median (range)**	**2 (0–6)**
**PARI risk category, *n* (%)**	​
Standard risk (score 0–2)	16 (55.2)
High risk (score ≥3)	13 (44.8)
**Donor, *n* (%)**	​
MRD	2 (6.9)
MUD	11 (37.9)
MMUD	12 (41.4)
Haplo	4 (13.8)
**HSC source, *n* (%)**	​
Apheresis	13 (44.8)
Bone marrow	14 (48.3)
Cord blood	2 (6.9)
**Conditioning regimen, *n* (%)**	​
Treo-Flu-TT	17 (58.6)
Treo-Flu	6 (20.7)
Flu-TT-Mel	2 (6.9)
Treo-Cy-Mel	1 (3.4)
Treo-Flu-Mel	1 (3.4)
Flu-TT	1 (3.4)
Flu	1 (3.4)
**GVHD prophylaxis, *n* (%)**	​
Thy-Tac-MTX	10 (34.5)
Thy-Csa-MTX	6 (20.7)
Thy-PTCy100-MMF-Csa	2 (6.9)
Alem-Csa-MTP	2 (6.9)
Alem-Csa-MMF	2 (6.9)
Thy-Csa-MTP	1 (3.4)
Thy-Tac-MTP	1 (3.4)
Tac-MTX	1 (3.4)
Thy-Csa-MMF	1 (3.4)
Thy-PTCy100-Csa	1 (3.4)
Thy-Tac-MTX-Rapamycin	1 (3.4)
Thy-PTCy100-MMF-Tac	1 (3.4)
**Serotherapy, *n* (%)**	​
Anti-thymocyte globulin	24 (82.8)
Alemtuzumab	4 (13.8)
None	1 (3.4)
**Conditioning type, *n* (%)**	​
MAC	20 (69)
RIC	9 (31)
**Donor chimerism at 1 year or last assessment, *n* (%)**	​
Full donor chimerism (>95%)	20 (74)
Partial chimerism (50–95%)	6 (22)
Low-level chimerism (5–50%)	1 (4)

IDDA, Immune Deficiency and Dysregulation Activity; HSCT, hematopoietic stem cell transplantation; MRD, matched related donor; MUD, matched unrelated donor; MMUD, mismatched unrelated donor; HSC, hematopoietic stem cell; Treo, treosulfan; Flu, fludarabine; Mel, melphalan; TT, thiotepa; Cy, cyclophosphamide; GVHD, graft-versus-host disease; Thy, anti-thymocyte globulin; Csa, cyclosporine; Tac, tacrolimus; MTX, methotrexate; MMF, mycophenolate mofetil; MTP, methylprednisolone; PTCy, posttransplant cyclophosphamide; Alem, alemtuzumab.

The median pretransplant IDDA v2.1 score was 8.5 (range 0–23.7). Pretransplant comorbidity burden assessed by HCT-CI classified the majority of patients as low (*n* = 12, 41.4%) or intermediate risk (*n* = 14, 48.3%), with only 3 patients (10.3%) classified as high risk; 89.7% of patients therefore fell in the low/intermediate categories, reflecting the recognized limitation of this index in pediatric populations. Using PARI, 13 patients (44.8%) were classified as high risk (score ≥3) and 16 patients (55.2%) as low risk (score <3). Group-level IDDA v2.1 scores are presented in Table 1, cohort HCT-CI and PARI distributions in Table 2, and individual-level score data in Tables S1 and S2.

Seven of 29 patients (24.1%) received bridging therapy prior to HSCT: cyclosporine A in four patients (SCID-DCLRE1C with Omenn phenotype, Wiskott–Aldrich syndrome [WAS] with hemophagocytic lymphohistiocytosis–like preconditioning manifestations, and two patients with SCID-RAG2 and maternal engraftment); rapamycin in two patients (APDS1 due to PIK3CD mutation with autoimmune cytopenias, and immune dysregulation, polyendocrinopathy, enteropathy, X-linked syndrome (IPEX syndrome) due to FOXP3 mutation with cutaneous and gastrointestinal manifestations); and ruxolitinib in one patient (STAT1 gain-of-function [STAT1-GOF]). Details are provided in Table S2.

### Transplant outcomes

**Figure 1.**
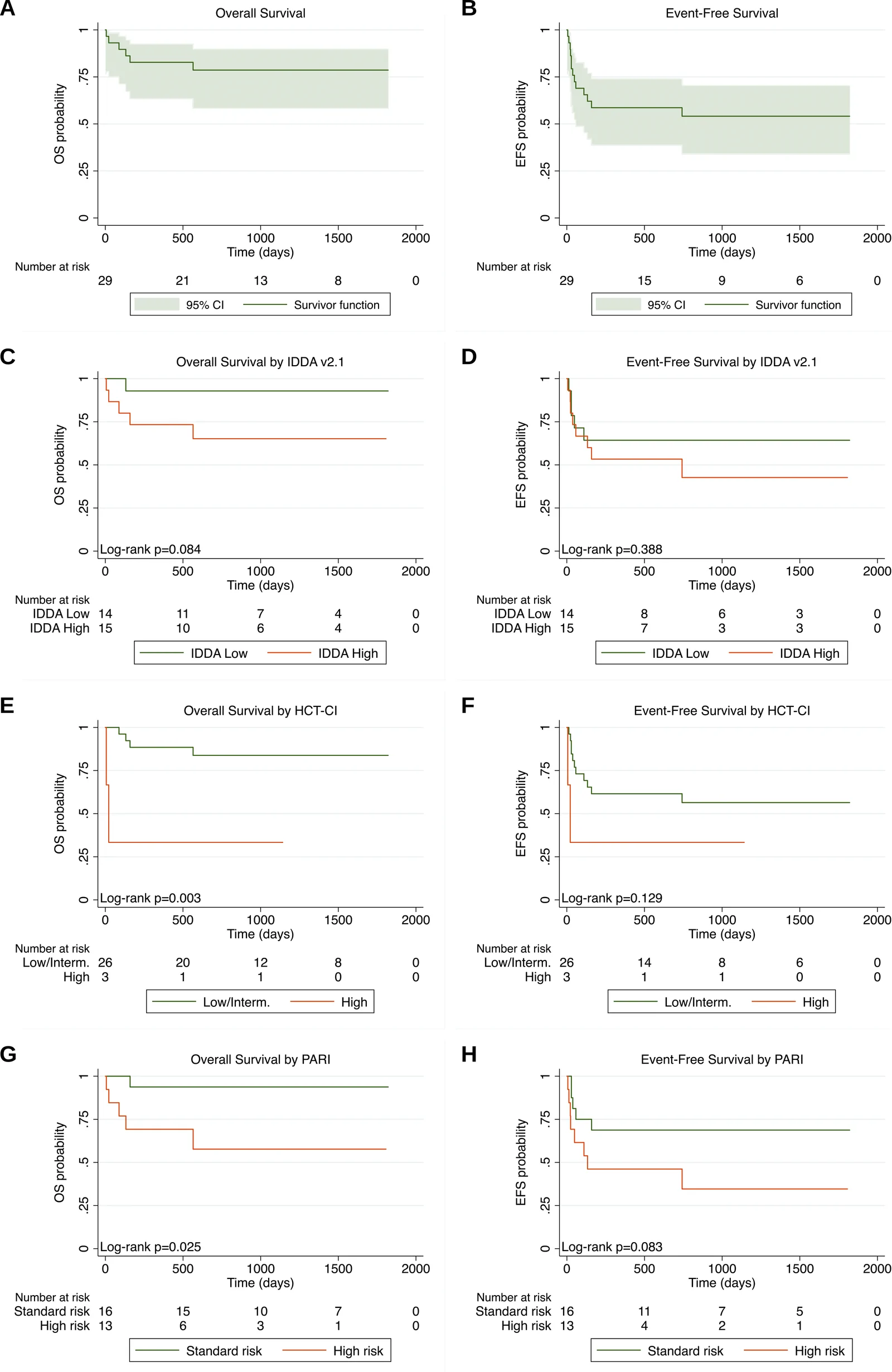
**Kaplan–Meier survival analyses after allogeneic HSCT in pediatric patients with IEI. (A)** OS in the study cohort. Estimated 2-year OS was 79% (95% CI: 58–90%). **(B)** EFS in the study cohort. Events were defined as acute GVHD grade ≥II, chronic GVHD, graft failure, or death. Estimated 2-year EFS was 59% (95% CI: 39–74%). **(C)** OS stratified by pretransplant IDDA v2.1 score (below vs. at/above cohort median of 8.5). **(D)** EFS stratified by pretransplant IDDA v2.1 score (below vs. at/above cohort median of 8.5). **(E)** OS stratified by HCT-CI category (<3 vs. ≥3). **(F)** EFS stratified by HCT-CI category (<3 vs. ≥3). **(G)** OS stratified by PARI risk category (<3 vs. ≥3). **(H)** EFS stratified by PARI risk category (<3 vs. ≥3). Number-at-risk tables are shown below each panel. OS, overall survival; EFS, event-free survival; HCT-CI, Hematopoietic Cell Transplantation–Specific Comorbidity Index; HSCT, hematopoietic stem cell transplantation; IDDA, Immune Deficiency and Dysregulation Activity; PARI, Pediatric Adapted Risk Index.

During the follow-up period, 6 deaths and 13 composite events were observed. The estimated 2-year OS for the entire cohort was 79% (95% CI: 58–90%), while the 2-year EFS was 59% (95% CI: 39–74%) (Fig. 1, A and B). In sensitivity analysis applying a stricter EFS definition that considered only acute GVHD grade ≥III as an event, 2-year EFS was 69% (95% CI: 49–82%).

Six deaths were observed in the cohort. One patient with WAS who underwent MUD cord blood transplantation died 22 days after HSCT due to acute respiratory distress syndrome (ARDS) and multi-organ failure. A second patient with WAS who received an MMUD cord blood transplant died 565 days after HSCT due to ARDS in the context of secondary graft failure and chronic CMV infection. A patient with STAT1-GOF who received a MMUD peripheral blood HSCT developed acute GVHD followed by transplant-associated thrombotic microangiopathy (TA-TMA), leading to death at day 132 after HSCT. Another patient with SCID due to CD3D deficiency who underwent haploidentical bone marrow transplantation died at day 6 after HSCT from ARDS and multi-organ failure. A patient with hyper-IgM syndrome due to CD40LG deficiency who underwent MMUD bone marrow transplantation developed acute GVHD and subsequently died at day 88 after HSCT due to ARDS. Finally, a patient with NFKB2 deficiency who received a MMUD bone marrow transplant died at day 159 after HSCT due to cerebral injury. A descriptive analysis of the leading IDDA v2.1 parameter domain (highest-scoring individual item) among the six patients who died revealed that the dominant manifestation domains were infection burden, hematologic/autoimmune involvement, and skin/mucocutaneous disease. The patient with the highest IDDA score in the cohort (SCID-CD3D, IDDA 23.7) had life-threatening refractory infections as the leading parameter (grade 4), with 39 days of mechanical ventilation in the pretransplant period, and died at day +6. The patient on ruxolitinib bridging at the time of assessment (STAT1-GOF, IDDA 3.5) had the lowest score among those who died, illustrating the potential underestimation of immune dysregulation burden in patients on active targeted therapy at the time of assessment. Full individual IDDA parameter grades for all 29 patients are provided in Table S1.

Regarding transplant-related complications, acute GVHD grades ≥II and ≥III occurred in eight patients (27.6%) and three patients (10.3%), respectively, while no cases of chronic GVHD were observed. Graft failure was documented in three patients (10.3%), and TA-TMA occurred in three patients (10.3%). No cases of sinusoidal obstruction syndrome (SOS) were reported.

### Association between IDDA v2.1 and transplant outcomes

When analyzed as a continuous variable, higher pretransplant IDDA v2.1 scores were significantly associated with worse OS in univariate Cox regression (six events; HR: 2.23 per five-point increase, 95% CI: 1.03–4.85, P = 0.043). The proportional hazards assumption was met. IDDA v2.1 remained associated with OS in an exploratory expanded multivariable model after adjustment for age at transplantation, donor type, conditioning intensity (MAC vs. RIC), stem cell source, and active infection at day 0 (HR: 2.76 per five-point increase, 95% CI: 1.04–7.31, P = 0.041) (Table 3). Given the ratio of six events to five covariates, the latter model should be interpreted with appropriate caution. In a separate model including IDDA v2.1, HCT-CI, and age, IDDA v2.1 did not retain statistical significance (P = 0.081), whereas HCT-CI remained associated with OS (HR: 1.75 per unit increase, P = 0.041), suggesting a partial overlap in the prognostic information captured by both instruments.

**Table 3.** Uni- and multivariate Cox regression analyses of pretransplant IDDA v2.1 score and post-HSCT outcomes

Outcome	Events	HR	95% CI	P value
OS (univariate analysis)	6	2.23	1.03–4.85	0.043
OS (multivariate analysis)	6	2.76	1.04–7.31	0.041
EFS	13	1.33	0.80–2.18	0.27
Acute GVHD (≥ grade II)	8	0.84	0.43–1.63	0.599
Graft failure	3	0.91	0.30–2.77	0.872
Thrombotic microangiopathy	3	0.80	0.26–2.44	0.70

The expanded exploratory multivariate model included IDDA v2.1, age at HSCT, donor type, conditioning intensity, stem cell source, and active infection at day 0. The joint model included IDDA v2.1, HCT-CI, and age. IDDA v2.1 HRs are expressed per five-point increase.

In contrast, no significant association was observed between IDDA v2.1 scores and EFS (HR: 1.33 per five-point increase, 95% CI: 0.80–2.18, P = 0.269) (Table 3). This finding remained unchanged in post hoc sensitivity analysis restricting acute GVHD events to grade ≥III (HR: 1.53 per five-point increase, 95% CI: 0.87–2.69, P = 0.141). Analysis of individual EFS components showed no significant association between IDDA v2.1 scores and acute GVHD grade ≥II (eight events; HR: 0.84, 95% CI: 0.43–1.63, P = 0.599) or graft failure (three events; HR: 0.91, 95% CI: 0.30–2.77, P = 0.872). Taken together, these analyses indicate that IDDA v2.1 predicts OS but not EFS in this cohort, irrespective of the GVHD grading threshold applied.

**Figure S1.**
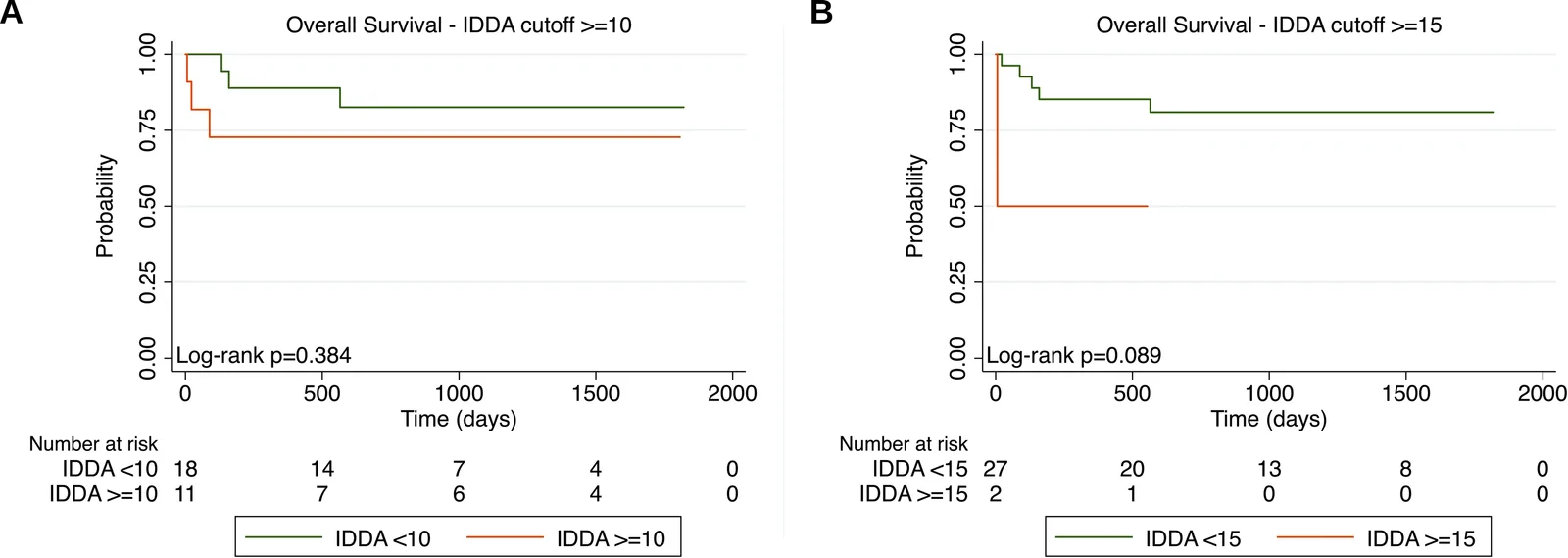
**Kaplan–Meier estimates of OS using alternative IDDA v2.1 cutoffs.** **(A)** Cutoff ≥10 (IDDA <10, *n* = 18; IDDA ≥10, *n* = 11; log-rank P = 0.384). **(B)** Cutoff ≥15 (IDDA <15, *n* = 27; IDDA ≥15, *n* = 2; log-rank P = 0.089). Number-at-risk tables are shown below each panel. OS, overall survival; IDDA, immune deficiency and dysregulation activity.

The Kaplan–Meier analysis stratified by the cohort median IDDA v2.1 score (8.5) showed inferior OS in patients with high pretransplant immune dysregulation, although this difference did not reach statistical significance in this small cohort (log-rank P = 0.084). No significant difference in EFS was observed between groups (log-rank P = 0.388) (Table 4 and Fig. 1, C and D). Kaplan–Meier analyses using alternative categorical cutoffs showed that neither IDDA v2.1 ≥ 10 (log-rank P = 0.384) nor IDDA v2.1 ≥ 15 (log-rank P = 0.089) achieved statistically significant OS separation, further supporting the continuous Cox model as the primary analytic approach (Fig. S1).

**Table 4.** Post-HSCT outcomes according to the IDDA v2.1 score (dichotomized at the median)

Outcome	IDDA < median (*n* = 14)	IDDA ≥ median (*n* = 15)	P value (Fisher’s test)
Death, *n* (%)	1 (7.1)	5 (33.3)	0.17
Acute GVHD ≥ II, *n* (%)	5 (35.7)	3 (20)	0.43
Acute GVHD ≥ III, *n* (%)	1 (7.1)	2 (13.3)	1
Chronic GVHD, *n* (%)	0 (0)	0 (0)	n/a
Graft failure, *n* (%)	1 (7.1)	2 (13.3)	1
Thrombotic microangiopathy, *n* (%)	2 (14.3)	1 (6.7)	0.6
SOS, *n* (%)	0 (0)	0 (0)	n/a

GVHD, graft-versus-host disease; n/a, not available.

### Comparative risk stratification: HCT-CI, PARI, and IDDA v2.1

When HCT-CI was analyzed as a continuous variable in univariate Cox regression, higher scores were significantly associated with worse OS (HR: 1.70 per unit increase, P = 0.009; C-statistic 0.661). Stratification by HCT-CI category (≥3 vs. <3) also revealed significantly inferior OS in the high-risk group (log-rank P = 0.003; C-statistic 0.661) (Fig. 1 E), whereas no significant difference was observed for EFS (log-rank P = 0.129) (Fig. 1 F). Stratification by PARI score (≥3 vs. <3) revealed significantly inferior OS in the high-risk group (log-rank P = 0.025; C-statistic 0.772) (Fig. 1 G), with a trend toward inferior EFS that did not reach statistical significance (log-rank P = 0.083) (Fig. 1 H). When PARI was analyzed as a continuous variable in univariate Cox regression, it was additionally associated with inferior EFS (HR: 1.35 per unit increase, 95% CI: 1.01–1.81, P = 0.042), in contrast to IDDA v2.1 and HCT-CI, which did not significantly predict EFS under any analytical approach. The C-statistics of IDDA v2.1 (0.722), HCT-CI (0.661), and PARI (0.772) were not statistically significantly different in this cohort, suggesting comparable discriminatory performance across the three instruments.

**Figure 2.**
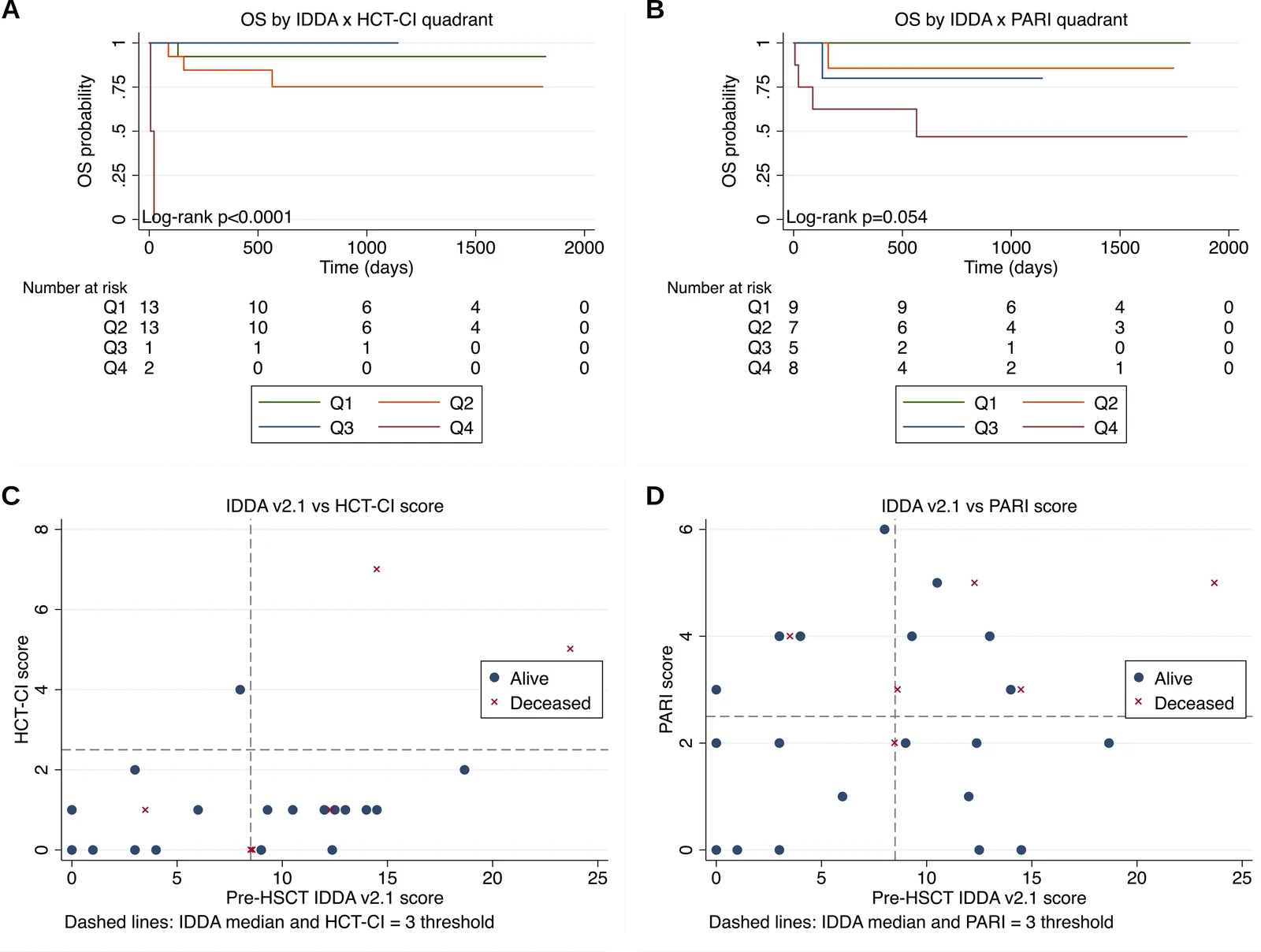
**Combined IDDA v2.1 and comorbidity score analyses. (A)** OS by combined IDDA v2.1 and HCT-CI quadrant: Q1, low HCT-CI/low IDDA (*n* = 13, 1 death); Q2, low HCT-CI/high IDDA (*n* = 13, 3 deaths); Q3, high HCT-CI/low IDDA (*n* = 1, 0 deaths); Q4, high HCT-CI/high IDDA (*n* = 2, 2 deaths). Log-rank P < 0.0001. **(B)** OS by combined IDDA v2.1 and PARI quadrant: Q1, standard PARI/low IDDA (*n* = 9, 0 deaths); Q2, standard PARI/high IDDA (*n* = 7, 1 death); Q3, high PARI/low IDDA (*n* = 5, 1 death); Q4, high PARI/high IDDA (*n* = 8, 4 deaths). Log-rank P = 0.054. **(C)** Scatter plot of IDDA v2.1 (x axis) versus HCT-CI score (y axis). Circles: alive at last follow-up; crosses: deceased. Dashed lines indicate cohort median IDDA (8.5) and HCT-CI high-risk threshold (≥3). **(D)** Scatter plot of IDDA v2.1 (x axis) versus PARI score (y axis). Circles: alive; crosses: deceased. Dashed lines indicate cohort median IDDA (8.5) and PARI high-risk threshold (≥3). Number-at-risk tables are shown below panels A–B. HCT-CI, Hematopoietic Cell Transplantation–Specific Comorbidity Index; IDDA, Immune Deficiency and Dysregulation Activity; OS, overall survival; PARI, Pediatric Adapted Risk Index.

An exploratory quadrant analysis combining IDDA v2.1 (dichotomized at the cohort median of 8.5) with HCT-CI category (≥3 vs. <3) identified four risk subgroups with significantly different OS (log-rank P < 0.0001) (Fig. 2, A and C). Patients with both low HCT-CI (<3) and low IDDA (*n* = 13) experienced one death (7.7%), compared with three deaths (23.1%) among those with low HCT-CI but high IDDA (*n* = 13). Among the smaller group with high HCT-CI (≥3), one patient had low IDDA (no deaths) and two had high IDDA (two deaths, 100%). This analysis suggests that IDDA v2.1 may identify high-risk patients within the predominant low/intermediate HCT-CI stratum, where conventional comorbidity scoring provides limited discrimination. A parallel quadrant analysis combining IDDA v2.1 with PARI score showed a trend toward differential OS across subgroups (log-rank P = 0.054) (Fig. 2, B and D). Among patients with PARI <3, those with low IDDA (*n* = 9) had no deaths, compared with one death among those with high IDDA (*n* = 7). Among patients with PARI ≥3, those with low IDDA (*n* = 5) had one death (20%), while those with both high PARI and high IDDA (*n* = 8) had four deaths (50%), suggesting a gradient of risk when both instruments are considered jointly.

### Peripheral blood chimerism

Posttransplant donor chimerism data were available for 27 patients. Chimerism assessed at 1 year or last follow-up demonstrated complete donor chimerism (>95%) in 20 of 27 patients (74%), while mixed chimerism was observed in seven patients (26%). Among those with mixed chimerism, six out of seven patients had donor chimerism between 50 and 95%, and only one patient had lower level donor chimerism between 5 and 50% (Table 2). No association was observed between pretransplant IDDA v2.1 scores and posttransplant chimerism status when comparing patients with complete versus mixed chimerism (P = 0.88). Chimerism was assessed using peripheral whole-blood samples. Importantly, none of the patients with mixed chimerism required donor lymphocyte infusion, and all maintained adequate disease control, suggesting sufficient functional engraftment despite the absence of lineage-specific chimerism data.

## Discussion

In this single-center cohort of pediatric patients with IEI undergoing allogeneic HSCT, higher pretransplant immune dysregulation measured by the IDDA v2.1 score was significantly associated with inferior OS. Each five-point increase in IDDA v2.1 was associated with a more than twofold increase in the hazard of death in univariate analysis and remained associated in an exploratory expanded model. These findings are consistent with those recently reported by Fox et al., who demonstrated that elevated pretransplant IDDA v2.1 scores are associated with poorer outcomes after HSCT in adolescents and adults with IEI (18), and extend the evaluation of IDDA v2.1 to an exclusively pediatric population. In their study, IDDA v2.1 significantly predicted EFS in univariate analysis, although its independent effect was attenuated in multivariable models, where the combination of high IDDA v2.1 and HCT-CI scores emerged as a strong predictor of both OS and EFS. Similar observations have been reported in disease-specific cohorts, where lower pretransplant IDDA scores were associated with improved overall and disease-free survival in patients with CTLA-4 deficiency undergoing HSCT (19). Likewise, Tesch et al. observed that lower pre-HSCT IDDA values were associated with better survival probability in LRBA deficiency, although these findings were limited by small sample size (14). Our results extend these observations to a pediatric-specific population, indicating that the burden of immune dysregulation prior to transplantation, as measured by the IDDA v2.1 score, may influence transplant-related mortality across different age groups and across diverse IEIs, supporting its relevance beyond disease-specific contexts. Comparative analysis with established comorbidity indices (HCT-CI and PARI) further showed that IDDA v2.1 provides complementary prognostic information, particularly within the low/intermediate HCT-CI stratum in which the majority of pediatric patients are classified.

Although the IDDA score was originally developed to quantify disease activity in patients with LRBA deficiency, the updated IDDA v2.1 score has increasingly been applied across a broader spectrum of IEIs to capture systemic immune dysregulation (14, 15, 19). In the present study, we therefore included patients with a wide range of genetically defined IEIs rather than restricting the analysis to PIRDs, reflecting the concept that immune dysregulation can occur across multiple categories of IEI, can be captured by the IDDA score, and may influence transplant outcomes irrespective of the underlying genetic diagnosis.

An important difference between our cohort and that reported by Fox et al. is the overall burden of immune dysregulation prior to transplantation. In their study of adolescents and adults with IEI, the median pretransplant IDDA v2.1 score was 16.6, whereas in our pediatric cohort, the median score was substantially lower at 8.5 (18). This difference may reflect earlier referral and transplantation in pediatric patients before the accumulation of long-standing inflammatory complications, autoimmunity, and organ damage that often develop over time in older patients with IEI (20, 21). Additionally, only one patient in our cohort belonged to the IUIS category of primary immune dysregulatory disorders, which may also have contributed to the relatively lower median IDDA v2.1 score. However, several other patients carried IEI with prominent immune dysregulatory manifestations, including STAT1-GOF, APDS1, WAS, CD40LG deficiency, and RAG2-deficient SCID presenting with Omenn syndrome or maternal engraftment. Despite this lower overall inflammatory burden, the association between higher IDDA v2.1 scores and increased mortality remained evident in our cohort. Unlike Fox et al., who derived an optimal IDDA v2.1 cutoff of 15 based on maximization of Harrell’s C-index, we did not attempt to define a data-driven threshold given the limited sample size and number of events, which could lead to overfitting. Larger studies are needed to establish and validate clinically meaningful cutoff values in pediatric populations.

In our cohort, the 2-year OS was 79% (95% CI: 58–90%) and the 2-year EFS was 59% (95% CI: 39–74%), consistent with outcomes reported from other experienced centers (11, 19, 22, 23, 24, 25, 26, 27, 28, 29, 30, 31, 32). EFS analyses in this cohort should be considered exploratory given the small number of events (*n* = 13) distributed across a heterogeneous event composite. The relatively low EFS likely reflects the broad event definition, which included acute GVHD grade ≥II. Acute GVHD grade II is often clinically manageable and may be transient (33, 34, 35). However, we deliberately included this threshold in order to better capture posttransplant immune complications and to explore their potential association with pretransplant immune dysregulation. When the sensitivity analysis was restricted to grade ≥III acute GVHD, IDDA v2.1 remained nonsignificantly associated with EFS (log-rank P = 0.162). The lack of association with EFS under any threshold may reflect limited statistical power, or may indicate that pretransplant immune dysregulation is more closely related to mortality than to intermediate transplant-related complications, which are also shaped by donor type, conditioning intensity, and posttransplant management factors beyond baseline inflammatory status.

Comparative analysis with established pretransplant risk tools showed that IDDA v2.1 (C-statistic 0.722) had discriminatory performance for OS comparable to HCT-CI (0.661) and PARI (0.772). A key finding of the quadrant analyses was that IDDA v2.1 stratified risk within the low/intermediate HCT-CI category—where 89.7% of our patients fell—identifying patients at substantially higher mortality who would otherwise appear low-risk by comorbidity scoring alone. This is clinically relevant given that HCT-CI was developed for adult populations and has recognized limitations in the pediatric setting, where most adult-defined comorbidities are absent (16). PARI, which was specifically designed for pediatric HSCT and showed the highest C-statistic in our cohort (0.772), is a promising complementary tool, though its validation in IEI-specific populations remains limited (17). The partial attenuation of the IDDA v2.1 signal when jointly modeled with HCT-CI (P = 0.081) likely reflects information overlap in this small cohort. Taken together, these findings support incorporating IDDA v2.1 alongside established indices rather than as a replacement, to improve pretransplant risk stratification in pediatric IEI patients.

Although a direct causal relationship between reduction of the IDDA v2.1 score and improved transplant outcomes cannot be established from these retrospective data, the score captures disease manifestations that are recognized targets for pretransplant optimization (6, 11, 36). Emerging evidence across different IEIs supports the use of bridging strategies to achieve disease control before transplantation—including JAK inhibitors in STAT1-GOF disease (37, 38), IFN-γ blockade with emapalumab in IFN-γ–driven inflammatory pathologies (39, 40, 41), and mTOR inhibition with rapamycin in Tregopathies such as IPEX syndrome (26, 42, 43)—though the benefit of specific interventions varies according to the underlying disease mechanism, as illustrated in CTLA-4 insufficiency where pretransplant abatacept did not significantly impact outcomes (19). These considerations underline the potential value of pretransplant IDDA v2.1 assessment in identifying patients who may benefit most from targeted disease control prior to HSCT, and in monitoring the adequacy of that response over time.

In our cohort, patients with mixed chimerism maintained adequate disease control without the need for additional cellular interventions, suggesting sufficient functional engraftment. This is consistent with previous reports in IEI, where stable mixed chimerism is frequently observed and may be compatible with long-term disease correction, in contrast to hematologic malignancies where complete donor chimerism is typically required for cure (9, 24, 44, 45).

This study has several limitations. First, it is a retrospective single-center analysis, subject to the inherent selection and ascertainment biases of this design. Second, the cohort is relatively small (*n* = 29), reflecting the rarity of pediatric IEI requiring HSCT, and the number of outcome events (six deaths) limits the reliability of multivariable models. Third, the cohort included a heterogeneous spectrum of IEI (19 distinct genetic entities), with relatively limited representation of patients classified within the IUIS category of primary immune dysregulatory disorders, which may have contributed to the overall lower IDDA v2.1 scores observed in this cohort and may limit generalizability to populations enriched for immune dysregulation phenotypes. Fourth, IDDA v2.1 scores were calculated retrospectively by a single investigator with consensus review; no formal inter-rater reliability coefficient (e.g., intraclass correlation) was prespecified, which is a methodological limitation that prospective studies should address. Fifth, in patients receiving targeted bridging therapy at the time of IDDA assessment (e.g., ruxolitinib for STAT1-GOF), the score may underestimate the true underlying burden of immune dysregulation, as illustrated by the patient with the lowest pretransplant score among those who died. Sixth, posttransplant chimerism was assessed on whole peripheral blood only; the absence of lineage-specific chimerism data limits interpretation of engraftment quality in this cohort. Lastly, the expanded multivariable model was exploratory and potentially unstable because only six deaths occurred. The quadrant and C-statistic analyses were likewise descriptive and cannot establish incremental prognostic value. In addition, longitudinal and posttransplant IDDA v2.1 scores could not be reconstructed at uniform predefined time points because documentation of all score components was not consistently available after HSCT. Despite these limitations, our findings provide the first pediatric-specific evidence linking pretransplant immune dysregulation quantified by IDDA v2.1 to transplant mortality, and demonstrate its potential value as a complementary risk stratification tool alongside HCT-CI and PARI.

### Conclusion

To our knowledge, this is the first study to evaluate the prognostic impact of IDDA v2.1 on transplant outcomes in an exclusively pediatric cohort of patients with IEI. Higher pretransplant IDDA v2.1 scores were independently associated with inferior OS across multiple analytical approaches, although the limited number of deaths restricts the strength of multivariable and comparative analyses. Exploratory comparison with established pretransplant risk scores showed that IDDA v2.1 may provide prognostic information complementary to HCT-CI and PARI, and is capable of identifying patients at elevated risk within the low/intermediate HCT-CI stratum in which the vast majority of pediatric patients are classified. Although these findings require validation in larger prospective cohorts, they support incorporating IDDA v2.1 alongside established risk indices to improve individualized pretransplant risk stratification and patient counseling in the pediatric HSCT setting. Prospective studies with longitudinal IDDA v2.1 assessment—including posttransplant reassessment and its correlation with immune reconstitution and chimerism—are warranted to further define the clinical utility of this tool in the pediatric HSCT setting.

## Materials and methods

### Study design

We conducted a retrospective single-center cohort study including all pediatric patients with IEI who underwent allogeneic HSCT at our institution between January 2021 and August 2025. Patients lacking sufficient clinical information to calculate the required scores would have been excluded; however, all 29 transplanted patients during the study period had complete clinical data available, and no patients were excluded on this basis. Data from institutional electronic medical records and the allogeneic HSCT database were retrospectively collected between December 2025 and February 2026, with follow-up censored on February 13, 2026.

Pretransplant comorbidity burden was assessed using the HCT-CI score, retrospectively calculated for all patients according to the original scoring criteria (16). The HCT-CI assigns points for 17 specific comorbidities and categorizes patients as low (score 0), intermediate (score 1–2), or high (score ≥3) risk. Pretransplant pediatric risk was additionally assessed using the PARI score, a recently validated composite tool developed specifically for risk stratification in children undergoing allogeneic HSCT, with scores ≥3 considered high risk (17). Pretransplant immune dysregulation was assessed using the IDDA v2.1 score, a validated composite measure of immune deficiency and dysregulation burden encompassing infectious, autoimmune, inflammatory, lymphoproliferative, and organ-specific manifestations, as well as supportive care requirements and performance status, with higher scores indicating more severe disease activity (14, 15). All three scores were retrospectively calculated according to their respective published methodologies using Microsoft Excel spreadsheets containing the original scoring formulae, based on each patient’s clinical status during the week immediately preceding initiation of conditioning. Scores were calculated by the principal investigator (A. Soffia) and independently reviewed by the senior author (C. Sotomayor); discrepancies were resolved by consensus. The indication for HSCT in each patient was determined based on current international guidelines (9), integrating the underlying genetic diagnosis and clinical phenotype, and in most cases was further reviewed by a national multidisciplinary expert panel convened by the Chilean Ministry of Health responsible for evaluating HSCT indications in patients with IEI. The primary outcome of interest was OS, defined as the time from HSCT to death from any cause or last follow-up. Secondary outcomes included EFS, defined as survival without any of the following events: acute GVHD grade ≥II, chronic GVHD, graft failure, or death. Given the limited number and heterogeneous nature of composite events, all EFS analyses were considered exploratory. A sensitivity analysis was additionally performed using a stricter EFS definition in which acute GVHD grade ≥III, rather than grade ≥II, was considered an event, while all other EFS components remained unchanged. Additional posttransplant complications recorded included TA-TMA and SOS. Median follow-up was estimated using the reverse Kaplan–Meier method.

Posttransplant donor chimerism was assessed by short tandem repeat analysis on whole peripheral blood. Lineage-specific chimerism (T cell and myeloid compartments) is not performed routinely at our institution and was therefore not available for this cohort.

The study was approved by the Scientific Ethics Committee for Health Sciences of the Pontificia Universidad Católica de Chile (CEC-Salud UC), study ID 260304004. The requirement for informed consent was waived by the Ethics Committee due to the retrospective nature of the study.

### Statistical analysis

OS and EFS were estimated using the Kaplan–Meier method. For descriptive survival analyses, patients were stratified according to pretransplant IDDA v2.1 scores dichotomized at the cohort median, and survival curves were compared using the log-rank test. Alternative IDDA v2.1 cutoffs (≥10 and ≥15) were also explored in Kaplan–Meier analyses. To further evaluate the association between immune dysregulation and transplant outcomes, Cox proportional hazards regression models were constructed with IDDA v2.1 analyzed as a continuous variable, with hazard ratios (HR) reported per five-point increase in score. Given the limited number of deaths, multivariable analyses were considered exploratory. An expanded Cox regression model included IDDA v2.1, age at transplantation, donor type, conditioning intensity (MAC vs. RIC), stem cell source, and active infection at day 0. A separate model included IDDA v2.1, HCT-CI, and age at transplantation. Quadrant analyses combined IDDA v2.1, dichotomized at the cohort median, with HCT-CI (<3 vs. ≥3) and, separately, PARI (<3 vs. ≥3); these analyses were exploratory, and OS was compared across groups using the log-rank test. Discriminatory performance of IDDA v2.1, HCT-CI, and PARI was compared using Harrell’s C-statistic. Given the small number of deaths, C-statistic estimates were interpreted descriptively and were not used to establish incremental prognostic value. Statistical significance was defined as a two-sided P value <0.05. All analyses were performed using Stata/MP 14.0 for Mac.

### Online supplemental material

Table S1 provides individual pretransplant IDDA v2.1 total scores and component-level grades for all 29 patients. Table S2 details individual patient, transplant, and pretransplant risk-score characteristics. Fig. S1 shows Kaplan–Meier estimates of OS using alternative IDDA v2.1 cutoffs (≥10 and ≥15).

## Supplementary Material

Table S1 shows pretransplant IDDA v2.1 total scores and individual component values for each patient.

Table S2 shows individual patient characteristics, transplant variables, and pretransplant risk scores.

## Data Availability

The datasets generated and/or analyzed during the current study are not publicly available due to institutional policies but are available from the corresponding author on reasonable request.
