# Supplementary material for: Pretransplant immune dysregulation and survival after HSCT in pediatric inborn errors of immunity
Source: J Hum Immun. 2026 Oct 19;3(1):e20260093. doi: 10.70962/jhi.20260093 (PMC13679611; doi:10.70962/jhi.20260093)
Supplement: Supplementary file 1 [file jhi_20260093_tables1.docx]

**Supplementary Table S1. Pre-transplant IDDA v2.1 total scores and individual component values for each patient.**

Each component is scored 0–4 (0: absent/inactive; 1: mild, transient, no treatment; 2: moderate, intermittent therapy; 3: severe, continuous therapy; 4: life-threatening, refractory/irreversible), except where otherwise indicated. Components N–P are continuous numeric values; Q is scored 0/2/3.

**Legend:** A: Autoimmune cytopenia. B: Hemophagocytic lymphohistiocytosis. C: Enteropathy/inflammatory bowel disease. D: Lymphoproliferation, splenomegaly, or hepatomegaly. E: Parenchymal lung disease (including LIP/GLILD). F: Skin or eye manifestations (eczema, uveitis, alopecia, vitiligo, or other). G: Endocrinopathy (insulin-dependent diabetes mellitus, thyroiditis, or other). H: Arthritis or other musculoskeletal manifestations. I: Autoimmune hepatitis, cholangitis, or pancreatitis. J: Glomerulonephritis, nephropathy, or tubulopathy. K: Neurologic manifestations. L: Faltering growth, malabsorption, or wasting. M: Severe or opportunistic infections (excluding chronic infestation). N: Karnofsky/Lansky Performance Scale (%). O: Hospitalization days out of the preceding 100 days (including day clinic; excluding ICU). P: ICU or mechanical ventilation days out of the preceding 100 days. Q: Immunoglobulin substitution therapy (0: none; 2: sporadic; 3: regular). R: Chronic or recurring infestation/infection (worst single pathogen scored). S: Other organ dysfunction or malady (cardiomyopathy, renal failure, allergies, or other). T: Nutrition/dietary status (0: normal; 1: disease-adjusted; 2: partial formula; 3: tube feeding/partial PN; 4: total PN).

| **Patient** | **IDDA v2.1 Score** | **IDDA v2.1 Individual Components** | | | | | | | | | | | | | | | | | | | |
| --- | --- | --- | --- | --- | --- | --- | --- | --- | --- | --- | --- | --- | --- | --- | --- | --- | --- | --- | --- | --- | --- |
|  |  | **A** | **B** | **C** | **D** | **E** | **F** | **G** | **H** | **I** | **J** | **K** | **L** | **M** | **N** | **O** | **P** | **Q** | **R** | **S** | **T** |
| **1** | 0 | 0 | 0 | 0 | 0 | 0 | 0 | 0 | 0 | 0 | 0 | 0 | 0 | 0 | 100 | 0 | 0 | 0 | 0 | 0 | 0 |
| **2** | 13 | 0 | 2 | 0 | 0 | 0 | 0 | 0 | 0 | 0 | 0 | 0 | 0 | 2 | 100 | 100 | 0 | 3 | 0 | 0 | 0 |
| **3** | 14.5 | 0 | 0 | 0 | 2 | 0 | 3 | 0 | 0 | 0 | 0 | 0 | 0 | 0 | 100 | 100 | 0 | 3 | 0 | 0 | 0 |
| **4** | 12.5 | 0 | 0 | 0 | 0 | 0 | 0 | 0 | 0 | 0 | 0 | 0 | 1 | 0 | 100 | 40 | 0 | 3 | 4 | 0 | 0 |
| **5** | 14.5 | 0 | 0 | 0 | 0 | 0 | 0 | 0 | 0 | 0 | 0 | 0 | 0 | 3 | 100 | 100 | 0 | 3 | 0 | 0 | 3 |
| **6** | 3.5 | 0 | 0 | 0 | 0 | 0 | 0 | 1 | 0 | 0 | 0 | 0 | 0 | 0 | 100 | 0 | 0 | 0 | 2 | 0 | 0 |
| **7** | 0 | 0 | 0 | 0 | 0 | 0 | 0 | 0 | 0 | 0 | 0 | 0 | 0 | 0 | 100 | 0 | 0 | 0 | 0 | 0 | 0 |
| **8** | 12 | 3 | 0 | 0 | 3 | 0 | 0 | 0 | 0 | 0 | 0 | 0 | 0 | 0 | 100 | 0 | 0 | 3 | 0 | 0 | 0 |
| **9** | 23.7 | 0 | 0 | 0 | 0 | 0 | 0 | 0 | 0 | 0 | 0 | 0 | 0 | 4 | 80 | 22 | 39 | 3 | 0 | 0 | 3 |
| **10** | 0 | 0 | 0 | 0 | 0 | 0 | 0 | 0 | 0 | 0 | 0 | 0 | 0 | 0 | 100 | 0 | 0 | 0 | 0 | 0 | 0 |
| **11** | 6 | 0 | 0 | 0 | 0 | 0 | 0 | 0 | 0 | 0 | 0 | 0 | 0 | 0 | 80 | 0 | 0 | 3 | 0 | 3 | 0 |
| **12** | 10.5 | 0 | 0 | 0 | 0 | 0 | 0 | 0 | 0 | 0 | 0 | 3 | 0 | 0 | 100 | 0 | 0 | 0 | 0 | 4 | 2 |
| **13** | 3 | 0 | 0 | 0 | 0 | 0 | 0 | 0 | 0 | 0 | 0 | 0 | 0 | 0 | 100 | 0 | 0 | 3 | 0 | 0 | 0 |
| **14** | 8.6 | 3 | 0 | 0 | 0 | 0 | 0 | 0 | 0 | 0 | 0 | 0 | 0 | 0 | 80 | 0 | 0 | 3 | 0 | 0 | 0 |
| **15** | 12.4 | 2 | 0 | 0 | 1 | 2 | 0 | 0 | 0 | 0 | 0 | 0 | 0 | 0 | 80 | 0 | 0 | 3 | 0 | 0 | 0 |
| **16** | 12.3 | 0 | 2 | 0 | 0 | 0 | 0 | 0 | 0 | 0 | 0 | 0 | 0 | 0 | 70 | 0 | 0 | 3 | 0 | 2 | 3 |
| **17** | 8 | 0 | 0 | 0 | 0 | 0 | 0 | 0 | 0 | 0 | 0 | 0 | 0 | 0 | 100 | 10 | 0 | 3 | 4 | 0 | 0 |
| **18** | 8.5 | 0 | 0 | 0 | 0 | 0 | 3 | 0 | 0 | 0 | 0 | 0 | 0 | 0 | 100 | 0 | 0 | 3 | 1 | 0 | 0 |
| **19** | 3.0 | 2 | 0 | 0 | 0 | 0 | 0 | 0 | 0 | 0 | 0 | 0 | 0 | 0 | 100 | 0 | 0 | 0 | 0 | 0 | 0 |
| **20** | 9.3 | 0 | 0 | 0 | 0 | 0 | 1 | 0 | 0 | 0 | 0 | 0 | 0 | 3 | 100 | 3 | 0 | 3 | 0 | 0 | 0 |
| **21** | 3 | 0 | 0 | 0 | 0 | 0 | 0 | 0 | 0 | 0 | 0 | 0 | 0 | 0 | 100 | 0 | 0 | 3 | 0 | 0 | 0 |
| **22** | 1 | 0 | 0 | 0 | 0 | 0 | 0 | 0 | 0 | 0 | 0 | 0 | 0 | 0 | 100 | 0 | 0 | 0 | 1 | 0 | 0 |
| **23** | 3 | 0 | 0 | 0 | 0 | 0 | 0 | 0 | 0 | 0 | 0 | 0 | 0 | 0 | 100 | 0 | 0 | 0 | 3 | 0 | 0 |
| **24** | 0 | 0 | 0 | 0 | 0 | 0 | 0 | 0 | 0 | 0 | 0 | 0 | 0 | 0 | 100 | 0 | 0 | 0 | 0 | 0 | 0 |
| **25** | 9 | 0 | 0 | 0 | 0 | 0 | 0 | 0 | 0 | 0 | 0 | 0 | 0 | 0 | 100 | 60 | 0 | 3 | 1 | 0 | 1 |
| **26** | 18.7 | 0 | 0 | 3 | 0 | 0 | 1 | 3 | 0 | 0 | 0 | 0 | 3 | 0 | 90 | 0 | 0 | 0 | 0 | 0 | 2 |
| **27** | 4 | 0 | 0 | 0 | 0 | 0 | 0 | 0 | 0 | 0 | 0 | 0 | 0 | 0 | 100 | 0 | 0 | 3 | 1 | 0 | 0 |
| **28** | 0 | 0 | 0 | 0 | 0 | 0 | 0 | 0 | 0 | 0 | 0 | 0 | 0 | 0 | 90 | 0 | 0 | 0 | 0 | 0 | 0 |
| **29** | 14 | 0 | 0 | 0 | 0 | 0 | 0 | 0 | 0 | 0 | 0 | 0 | 0 | 0 | 100 | 100 | 0 | 3 | 4 | 0 | 3 |
