# Supplementary material for: Pretransplant immune dysregulation and survival after HSCT in pediatric inborn errors of immunity
Source: J Hum Immun. 2026 Oct 19;3(1):e20260093. doi: 10.70962/jhi.20260093 (PMC13679611; doi:10.70962/jhi.20260093)
Supplement: Supplementary file 2 [file jhi_20260093_tables2.docx]

**Supplementary Table S2. Individual patient characteristics, transplant variables, and pre-transplant risk scores.**

**Legend:** Donor matching: MRD, matched related donor; MMUD, mismatched unrelated donor; MUD, matched unrelated donor; Haplo, haploidentical donor. Stem cell source: BM, bone marrow; PB, peripheral blood; UCB, umbilical cord blood. Conditioning: MAC, myeloablative conditioning; RIC, reduced-intensity conditioning. Active infection at HSCT: presence of any clinically active infection at time of transplant. Bridging therapy: immunosuppressive or targeted therapy administered prior to HSCT to control disease activity (CSA, ciclosporin A; Rapamycin, sirolimus; Ruxolitinib). OS, overall survival from HSCT to last follow-up or death (days). Deceased: all-cause mortality at last follow-up. IDDA v2.1, Immune Deficiency and Dysregulation Activity score version 2.1. HCT-CI, Hematopoietic Cell Transplantation–Comorbidity Index (Sorror et al., 2005). PARI, Paediatric Aggregate Risk Index (Elfeky et al., 2024).

| **Patient** | **Diagnosis** | **Gene** | **Sex** | **Age at HSCT (years)** | **Donor matching** | **Stem cell source** | **Conditioning** | **Active infection at HSCT** | **Pre-HSCT Bridging Therapy** | **OS (days)** | **Deceased** | **IDDA v2.1 score** | **HCT-CI score** | **PARI score** |
| --- | --- | --- | --- | --- | --- | --- | --- | --- | --- | --- | --- | --- | --- | --- |
| **1** | GATA2 Def | GATA2 | M | 11.9 | MRD | BM | MAC | No | No | 1821 | No | 0 | 1 | 0 |
| **2** | SCID | DCLRE1C | M | 0.7 | MMUD | PB | RIC | Yes | CSA | 1807 | No | 13 | 1 | 4 |
| **3** | WAS | WAS | F | 1.4 | MUD | UCB | MAC | Yes | CSA | 22 | Yes | 14.5 | 7 | 3 |
| **4** | SCID | RAG2 | M | 0.7 | MUD | BM | RIC | Yes | CSA | 1746 | No | 12.5 | 1 | 0 |
| **5** | SCID | IL7R | M | 0.6 | MUD | PB | RIC | Yes | No | 1739 | No | 14.5 | 1 | 0 |
| **6** | STAT1-GOF | STAT1 | F | 5.5 | MMUD | PB | MAC | No | Ruxolitinib | 132 | Yes | 3.5 | 1 | 4 |
| **7** | LAD1 | ITGB2 | M | 2.1 | MMUD | PB | MAC | No | No | 1618 | No | 0 | 0 | 2 |
| **8** | APDS1 | PIK3CD | M | 5.8 | MUD | PB | MAC | No | Rapamycin | 1585 | No | 12 | 1 | 1 |
| **9** | SCID | CD3D | M | 0.3 | Haplo | BM | RIC | Yes | No | 6 | Yes | 23.7 | 5 | 5 |
| **10** | Neutropenia | ELANE | F | 12.2 | MUD | BM | MAC | No | No | 1527 | No | 0 | 0 | 0 |
| **11** | HIGM | CD40LG | M | 7.4 | MUD | PB | MAC | No | No | 1514 | No | 6 | 1 | 1 |
| **12** | CGD | CYBB | M | 10.2 | MMUD | PB | MAC | No | No | 1486 | No | 10.5 | 1 | 5 |
| **13** | HIGM | CD40LG | M | 17.7 | MUD | PB | MAC | No | No | 1451 | No | 3 | 0 | 0 |
| **14** | WAS | WAS | M | 1.7 | MMUD | UCB | RIC | No | No | 565 | Yes | 8.6 | 0 | 3 |
| **15** | HIGM | CD40LG | M | 8.7 | MUD | PB | MAC | No | No | 1186 | No | 12.4 | 0 | 2 |
| **16** | HIGM | CD40LG | M | 10.6 | MMUD | BM | MAC | Yes | No | 88 | Yes | 12.3 | 1 | 5 |
| **17** | SCID | ADA | F | 1.2 | Haplo | BM | RIC | Yes | No | 1142 | No | 8 | 4 | 6 |
| **18** | NFKB2 Def | NFKB2 | M | 10.2 | MMUD | BM | MAC | No | No | 159 | Yes | 8.5 | 0 | 2 |
| **19** | DKC | TERT | F | 5.8 | MUD | BM | RIC | No | No | 1059 | No | 3.0 | 0 | 0 |
| **20** | DOCK2 Def | DOCK2 | F | 3.3 | MMUD | BM | MAC | Yes | No | 910 | No | 9.3 | 1 | 4 |
| **21** | HIGM | CD40LG | M | 7.5 | MUD | PB | MAC | Yes | No | 842 | No | 3 | 2 | 4 |
| **22** | DKC | TINF2 | M | 13.5 | MRD | BM | RIC | No | No | 805 | No | 1 | 0 | 0 |
| **23** | GATA2 Def | GATA2 | F | 5.9 | MMUD | BM | MAC | No | No | 751 | No | 3 | 0 | 2 |
| **24** | GATA2 Def | GATA2 | F | 8.7 | MMUD | BM | MAC | No | No | 589 | No | 0 | 0 | 2 |
| **25** | SCID | RAG2 | F | 0.5 | Haplo | BM | RIC | No | CSA | 569 | No | 9 | 0 | 2 |
| **26** | IPEX | FOXP3 | M | 1.7 | MMUD | BM | MAC | No | Rapamycin | 554 | No | 18.7 | 2 | 2 |
| **27** | HIGM | CD40LG | M | 7.9 | MMUD | PB | MAC | Yes | No | 478 | No | 4 | 0 | 4 |
| **28** | WAS | WAS | M | 10.4 | Haplo | PB | MAC | Yes | No | 175 | No | 0 | 1 | 3 |
| **29** | SCID | LAT | F | 0.8 | MUD | PB | MAC | Yes | No | 169 | No | 14 | 1 | 3 |
